# Repurposing beta-3 adrenergic receptor agonists for Alzheimer’s disease: beneficial effects in a mouse model

**DOI:** 10.1186/s13195-021-00842-3

**Published:** 2021-05-21

**Authors:** Marine Tournissac, Tra-My Vu, Nika Vrabic, Clara Hozer, Cyntia Tremblay, Koralie Mélançon, Emmanuel Planel, Fabien Pifferi, Frédéric Calon

**Affiliations:** 1 Faculté de pharmacie, Université Laval, 1050 Avenue de la Médecine, Quebec, QC G1V 0A6 Canada; 2 Axe Neurosciences, Centre de recherche du CHU de Québec-Université Laval (Pavillon CHUL), 2705 Boulevard Laurier, Quebec, QC G1V 4G2 Canada; 3 UMR CNRS/MNHN 7179, Mécanismes Adaptatifs et Évolution, 1 Avenue du Petit Château, 91800 Brunoy, France; 4 Département de psychiatrie et neurosciences, Faculté de médecine, Université Laval, 1050 Avenue de la Médecine, Quebec, QC G1V 0A6 Canada

**Keywords:** Alzheimer’s disease, β3 adrenergic receptors, Drug repurposing, Thermogenesis, 3xTg-AD mice, Brown adipose tissue

## Abstract

**Background:**

Old age, the most important risk factor for Alzheimer’s disease (AD), is associated with thermoregulatory deficits. Brown adipose tissue (BAT) is the main thermogenic driver in mammals and its stimulation, through β3 adrenergic receptor (β3AR) agonists or cold acclimation, counteracts metabolic deficits in rodents and humans. Studies in animal models show that AD neuropathology leads to thermoregulatory deficits, and cold-induced tau hyperphosphorylation is prevented by BAT stimulation through cold acclimation. Since metabolic disorders and AD share strong pathogenic links, we hypothesized that BAT stimulation through a β3AR agonist could exert benefits in AD as well.

**Methods:**

CL-316,243, a specific β3AR agonist, was administered to the triple transgenic mouse model of AD (3xTg-AD) and non-transgenic controls from 15 to 16 months of age at a dose of 1 mg/kg/day i.p.

**Results:**

Here, we show that β3AR agonist administration decreased body weight and improved peripheral glucose metabolism and BAT thermogenesis in both non-transgenic and 3xTg-AD mice. One-month treatment with a β3AR agonist increased recognition index by 19% in 16-month-old 3xTg-AD mice compared to pre-treatment (14-month-old). Locomotion, anxiety, and tau pathology were not modified. Finally, insoluble Aβ42/Aβ40 ratio was decreased by 27% in the hippocampus of CL-316,243-injected 3xTg-AD mice.

**Conclusions:**

Overall, our results indicate that β3AR stimulation reverses memory deficits and shifts downward the insoluble Aβ42/Aβ40 ratio in 16-month-old 3xTg-AD mice. As β3AR agonists are being clinically developed for metabolic disorders, repurposing them in AD could be a valuable therapeutic strategy.

**Supplementary Information:**

The online version contains supplementary material available at 10.1186/s13195-021-00842-3.

## Background

Old age is the main risk factor of Alzheimer’s disease (AD), a neurodegenerative disorder clinically expressed by memory deficits and cognitive dysfunction [1, 2]. The prevalence of AD is growing fast along with the aging population [3]. Yet, the exact pathogenic causes of the sporadic form of the disease are unknown. Despite decades of intense research and clinical trials, there is still no curative treatment for AD. Since AD is a complex and multifactorial disease, with frequent age-related comorbidities, multi-target agents might be advantageous over a single-bullet approach. The undeniable impact of old age on AD incidence indicates that aging triggers etiopathological factors of AD; identifying these key factors could provide invaluable clues to the development of novel therapeutic treatments.

Deficits in thermoregulation are among the documented consequences of old age. Although few studies investigated thermoregulation in AD individuals, it is well known that thermoregulatory defects appear in the elderly, the population primarily affected by AD [4–7]. Mounting evidence now supports the hypothesis that thermoregulation deficits contribute to the development of AD pathology. Spontaneous thermoregulation deficits occur in mouse models of AD neuropathology, including the triple transgenic (3xTg-AD) mice [8–11]. Studies in mouse and hibernators repeatedly showed that decreased body temperature leads to increased tau phosphorylation [12–14]. Supporting the link with age, cold-induced tau phosphorylation is potentiated in old mice compared to young mice [15]. Accordingly, acute manipulation of body temperature leads to strong modulation of AD neuropathology in mice: hypothermia induced by cold exposure increases both tau phosphorylation and amyloid-β (Aβ) pathology and decreases synaptic proteins, while restoring normothermia by exposure to higher room temperature reverses memory and anxiety-like behavior and reduced Aβ42 peptide levels in 3xTg-AD mice [9]. More recently, our group provided evidence that sustained enhancement of thermogenesis through cold acclimation improves metabolic disorders and protects old 3xTg-AD mice from cold-induced tau phosphorylation [16]. Altogether, these observations suggest that thermoregulatory mechanisms could be a potential therapeutic target in AD.

Beside thermoregulation, metabolic diseases share strong pathogenic links with AD. Indeed, induction of a diabetic phenotype such as glucose intolerance has been repeatedly shown to increase AD neuropathology in a mouse model of AD [17–20]. Central insulin signaling defects and lower brain glucose metabolism are observed in AD [21, 22]. It is estimated that one out of ten cases of AD is attributable to type 2 diabetes (T2D) [23]. These observations logically led to the idea of repurposing T2D drugs in AD [24]. Insulin, thiazolidinediones and glucagon-like peptide-1 analog are still the subject of clinical trials in dementia, albeit with mitigated results [25–27]. Thus, common metabolic targets between both diseases such as thermoregulatory defects are of interest to develop new therapeutic tools in AD.

Brown adipose tissue (BAT) is an essential thermogenic driver in mammals [28]. The discovery of functional BAT in adults in 2009 has revived research on this tissue [29, 30]. The ability of BAT thermogenesis to improve main metabolic disorders is now well-established in young [31–33] and old mice [16]. Pharmacological tools have been developed in this direction. In particular, β3 adrenergic receptor (β3AR) agonists are being extensively used to stimulate β3AR located on brown adipocytes, thereby leading to lipolysis and uncoupling protein 1 (UCP1) expression, the main marker of non-shivering thermogenesis [34, 35]. CL-316,243 is a highly specific β3AR agonist frequently used in metabolic studies in rodents. It has been shown to improve blood glucose metabolism, insulin sensitivity, and energy expenditure and to regulate lipids metabolism [36–40]. Since β3AR agonists can correct metabolic disorders by enhancing BAT activity, they could tackle both T2D and AD at the same time. Importantly, β3AR agonists have been shown to stimulate BAT activity in humans [41, 42], and one of these molecules, mirabegron (Myrbetriq®), is now approved for the treatment of overactive bladder [43]. Therefore, β3AR agonists could rapidly be tested in humans for dementia.

We hypothesized that pharmacological stimulation of BAT thermogenesis through β3AR agonist treatment could curtail AD neuropathology and improve memory as well as correcting thermoregulatory and metabolic deficits. To verify this hypothesis, 15-month-old non-transgenic (NonTg) and 3xTg-AD mice received daily CL-316,243 (1 mg/kg) or saline injections for a month.

## Methods

### Animals

The triple transgenic mouse model of AD (homozygous 3xTg-AD; APP_swe_, PS1_M146V_, tau_P301L_) developing both amyloid and tau pathologies in the brain with age was used here [44], and compared to a NonTg control mouse on the same genetic background (C57BL6/129Svj). We selected 15-month-old 3xTg-AD and NonTg controls, at an age when 3xTg-AD mice have extended plaques and tangles in the brain, as well as cognitive deficits [45–48]. Animals were produced at our animal facility and all maintained in the same genetic background (C57BL6/129SvJ) by backcrossing every 8–10 generations. Forty-two (42) mice were used for all experiments (*n* = 9–12 mice per group) and 9 mice were added for behavioral and glucose tolerance tests for a total of 51 mice (*n* = 9–16 mice per group).

Mice were housed one to five per cage at a housing temperature of 23.029 ± 0.098 °C, with a 12:12-h light-dark cycle (light phase from 7 a.m. to 7 p.m). Animals had ad libitum access to water and chow (Teklad 2018, Harlan Laboratories, Canada). Only males were used here to avoid temperature variation induced by the estrous cycle of female mice [49]. Food consumption was evaluated by weighing the diet of each cage and averaged for each mouse per day per cage every 4 days during the 1-month treatment, and 3 weeks before the beginning of the experiment. At the end of the experiment, all mice were put under deep anesthesia with ketamine/xylazine intraperitoneal (i.p.) injection (100 mg/kg ketamine, 10 mg/kg xylazine) and immediately placed under a heating pad to maintain body temperature until complete loss of posterior paw reflex. Then, mice were rapidly sacrificed by intracardiac perfusion with 0.1 M phosphate buffer saline (PBS) solution containing phosphatase (sodium pyrophosphate, 1 mM and sodium fluoride, 50 mM) and protease (Sigmafast protease inhibitor tablets, Sigma-Aldrich, St-Louis, USA) inhibitors. All experiments were performed in accordance with the Canadian Council on Animal Care and were approved by the Institutional Committee of the Centre Hospitalier de l’Université Laval (CHUL).

### CL-316,243 treatment

**Fig. 1:**
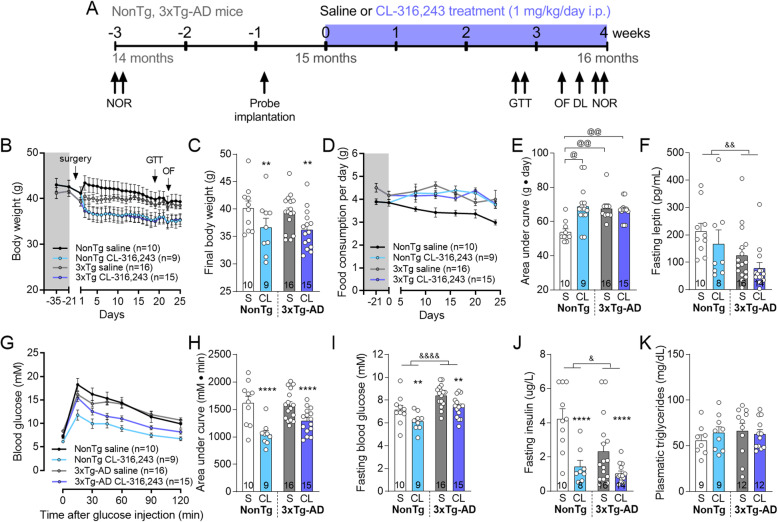
β3AR stimulation improves peripheral glucose metabolism in NonTg and 3xTg-AD mice. **a** Schematic description of the experimentation. **b** Body weight of the mice measured before and during the 1-month experiment (before each i.p. injection) and **c** at the end of the experiment. **d** Food consumed per day per mice 3 weeks before and over the 1-month treatment and **e** area under curve of food consumption over the 1-month treatment measured by weighting the diet every 4 days. **f** Leptin measured in plasma of fasted mice by ELISA. **g** GTT consists in measuring the blood glucose in fasted mice for 6-h after a bolus injection of glucose (1 g/kg i.p.). GTT was performed after 3 weeks of experiment and **h** area under curve of the GTT. **i** Fasting blood glucose and **j** plasmatic insulin measured during the GTT with a glucometer or by ELISA, respectively. **i** Triglycerides measured in the plasma sampled at the end of the experiment. Data are represented as mean ± SEM (*n*/group indicated in graphs). Statistics: Two-way ANOVA, effect of treatment: **p < 0.01; ****p < 0.0001; effect of genotype: ^&^*p* < 0.05; ^&&^*p* < 0.01; ^&&&&^*p* < 0.0001 (**c**, **f**, **h**, **i**, **j**). Kruskal-Wallis, Dunn’s post hoc test: ^@^*p* < 0.05; ^@@^*p* < 0.01 (**e**). Abbreviations: 3xTg-AD: triple transgenic mice; CL: CL-316,243-injected group; DL: dark-light emergence test; GTT: glucose tolerance test; NonTg: non-transgenic mice; NOR: novel object recognition test; OF: open field; S: saline-injected group

CL-316,243 was selected to stimulate BAT thermogenesis because it is one of the most selective β3AR agonists in rodents (β1: β2: β3 = 0:1:100,000) and its safety and efficacy has been confirmed in multiple studies [50–54]. Two to 3 weeks of daily injection at a dose of 1 mg/kg per day are necessary to improve metabolic disorders [36–40]. Thus, mice were injected i.p. every day for a month (25 consecutive injections) with a weight-adjusted dose of CL-316,243 (1 mg/kg) or an equivalent volume of saline (the vehicle) at the same hour of the day (4 p.m.) from 15 to 16 months of age (Fig. 1a). Mice were weighed every day before each i.p. injection. Mice were sacrificed the morning after the last injection (exactly on the 26th day after the first injection).

### Body temperature measurement and analysis

Telemetric probes (Anipill, Caen, France) were used to record body temperature of the animals every hour during the 1-month experiment without manipulation. Probes were implanted in the intraperitoneal cavity under isoflurane anesthesia a week before the beginning of the treatment to allow recovery from the surgery (Fig. 1a). Heat pads were used throughout the procedure to avoid hypothermia. The time under anesthesia was similar between mice and lasted approximately 10 min. Then, the animals were kept under heat pads during the waking period. Body temperature was analyzed during the two first weeks of treatment, before animals underwent glucose tolerance and behavioral tests, to avoid resulting interference in circadian rhythms.

In order to assess potential phase advances or delays and to visualize endogenous rhythmicity and regularity of the mice circadian clock, Clocklab software (Actimetrics Inc., Evanston, Illinois, USA) provided the further information: individual mean daily offsets in hours (determined as the time of the first six successive bins when temperature was lower than the mean diurnal temperature), offset standard deviation in hours and mean duration of a total temperature cycle in hours. We performed our analyses based on daily offsets (the end of the active period of the mice) because daily injections of the drug were performed only 3 h before the lights turned off, thus influencing the onsets (the beginning of the active period). Other following parameters were calculated for each individual: mean body temperature in degrees Celsius during the dark (from 7 p.m. to 7 a.m.) and light phase (from 7 a.m. to 7 p.m.) and mean amplitude of temperature (T°max − T°min) in degrees Celsius during the dark and light phase.

### Glucose tolerance test, fasting blood glucose, leptin, insulin, and triglyceride measurement

Glucose tolerance test (GTT) was performed at the end of the third week of treatment (Fig. 1a) [47]. Mice were fasted for 6 h (from 8 a.m. to 2 p.m.). Then, glucose was injected i.p. at 1 g/kg and blood glucose was measured regularly during 2 h with a glucometer (OneTouch UltraMini; LifeScan, Milpitas, CA) in a blood drop sampled from the saphenous vein. Leptin and insulin were assessed by ELISAs (Leptin Mouse ELISA kit, ab100718, Abcam; mouse insulin ELISA, #10-1247-01, Mercodia, Sweden) following the manufacturer’s instructions in plasma sampled in the saphenous vein after the 6-h fasting during the GTT test. Triglycerides were measured in blood sampled at the end of the experiment, just before sacrifice, by an enzymatic assay (Infinity Triglycerides Liquid Stable Reagent, Thermo Fisher Scientific, Waltham, MA).

### Behavioral tests

Behavioral tests were performed during the fourth week of treatment with a recovery time of at least 24 h between tests (Fig. 1a). The novel object recognition (NOR) test was also performed 3 weeks before the beginning of the treatment to obtain a baseline index ratio for each animal (see below). Mice were acclimated overnight to the testing room located next to the housing room.

Locomotor activity was assessed with the open field test [55]. Mice were placed in a 40 cm × 40 cm × 40 cm translucent Plexiglas box for an hour. Movements were tracked with photobeam breaks (San Diego Instruments). The total distance traveled (voluntary horizontal movement) and the average speed were compared between groups.

Anxiety behavior was evaluated with the dark-light emergence test [9]. Mice were put in the center of the dark compartment with an opening to the light compartment. The time spent in the light compartment and the latency to do the first exploration (nose latency) of the light compartment were measured during a 5-min trial.

Memory deficits were evaluated with the NOR test. That test detects behavioral deficits from 12 months in 3xTg-AD mice and is one of the less stressful behavioral test [9, 46, 56, 57]. It evaluates recognition memory and corresponds to episodic memory that is early affected in AD [58–60]. Mice were first placed in a 29.2 cm × 19 cm × 12.7 cm cage with two identical objects for 5 min during the acquisition phase. After an hour in their housing cage, mice returned in the testing cage containing a familiar and a novel object for the test phase. Recognition index (RI) corresponds to the time spent exploring the novel object divided by the total time of exploration during the test phase multiplied by 100. A 50% RI corresponds to an equal exploration between the novel and the familiar object. Mice exploring less than 6 s each object during the acquisition phase or less than 4 s during the test phase were excluded from the RI analysis. Mice were assigned to the treated or the control group at 15 months of age with caution to homogenize memory performance (baseline RI) between groups at the beginning of the experiment.

### Tissue preparation for postmortem analysis

Intracardiac blood sampled just before intracardiac perfusion in a heparinized tub was centrifuged at 3000 rpm for 5 min, and resulting plasma kept frozen at − 80 °C until analysis. The first hemisphere and interscapular BAT were rapidly dissected and frozen at − 80 °C until processing. The second hemisphere was either fixed in 4% paraformaldehyde for 48 h and transferred in a 20% sucrose solution until sectioning (3–4 mice per group) or frozen and kept at − 80 °C.

### Protein extractions

For the hippocampus, frozen samples were homogenized in 8 volumes of a lysis buffer (150 mM NaCl, 10 mM NaH_2_PO_4_, 0.5% sodium deoxycholate, 0.5% sodium dodecyl sulfate, 1% Triton X-100) containing a cocktail of protease and phosphatase inhibitors (Bimake, Houston, TX), sonicated (3 × 45 s in a Sonic Dismembrator apparatus, Thermo Fisher Scientific, Waltham, MA) and centrifuged (100,000*g*, 20 min, 4 °C), resulting in a detergent-soluble fraction (cytosolic, extracellular, and membrane-bound proteins). The remaining pellets from ultracentrifugation were resuspended in formic acid, resulting in a detergent-insoluble fraction (insoluble proteins fraction). The resultant suspension was sonicated and centrifuged (13,000*g*, 20 min, 4 °C), acid formic was evaporated and proteins were either solubilized in Laemmli’s buffer for Western blot or in a 5 M guanidium solution in Tris-HCl 50 mM for Aβ peptide ELISAs as previously described [61]. Proteins from the BAT were extracted in the lysis buffer only. Protein concentrations were evaluated with a bicinchoninic acid assay (BCA, Pierce, Rockford, IL, USA).

### Western immunoblotting

In total, 15 μg and 10 μg of proteins of hippocampus and BAT homogenates, respectively, were loaded and separated by SDS-PAGE, as previously described [17]. The list of antibodies used in this study is available in Additional File 3. Homogenates were all run on the same gel for each experiment. Membranes were imaged using the myECL imager system (Thermo Fisher Scientific). Quantifications were performed using the ImageLab software (Millipore), and the results were expressed as relative optical densities (OD). For the analysis of the protein tau, bands from all isoforms detected around 60 kDa were selected and quantified together.

### Aβ40 and Aβ42 peptides quantification

Aβ peptides were quantified in protein extracts from the hippocampus. Aβ40 and Aβ42 were measured in detergent-soluble and detergent-insoluble fractions using a human amyloid-β ELISA (Wako, Osaka, Japan) according to the manufacturer’s instructions. Plates were read at 450 nm using a Synergy^TM^ HT multi-detection microplate reader (Biotek, Winooski, VT).

### High-performance liquid chromatography (HPLC)

HPLC was used to measure the level of norepinephrine in BAT. An average of 10 mg of BAT was homogenized in perchloric acid (0.1 N) and centrifuged 10 min at 4 °C at 13,000 rpm. Five microliters of the supernatant was injected in the HPLC with electrochemical detection (Water 717 plus Autosampler automatic injector, Waters 1525 Binary Pump) as previously described [62].

### Statistical analysis

Data are represented as means ± standard error of the mean (SEM). Statistical analysis and the number of samples per group are specified in each figure and legend. Bartlett’s tests were used to rule out the inequality of variances between the groups. Two-way ANOVA (two independent variables: genotype and treatment) was used in case of equal variances. Repeated measures two-way ANOVA was executed to compare recurrent measurements in same animals. Tukey’s test was used for post hoc analysis. In case of unequal variances, a Kruskal-Wallis followed by a Dunn’s post hoc test was performed. An unpaired Student’s *t* test was performed when only two groups were compared, with a Welch correction in case of unequal variances. Paired *t* test was executed for the before-and-after on comparison on the same animals. One sample *t* test was used to compare means to a theoretical value (for the NOR test). Correlations between variables were investigated using linear regression analyses. A Grubb’s test was performed to test for outliers. All statistical analyses were performed with Prism 7 (GraphPad Software Inc., San Diego, CA, USA) or JMP (version 13.2.0; SAS Institute Inc., Cary, IL, USA) software and statistical significance was set at *p* < 0.05.

## Results

### β3AR stimulation improves peripheral glucose metabolism in old mice

NonTg and 3xTg-AD mice received CL-316,243 or saline i.p. at a dose of 1 mg/kg every day for a month from 15 to 16 months of age (Fig. 1a). To verify whether CL-316,243 affects energy balance, mice were weighed every day before each i.p. injection and food consumption was evaluated 3 weeks before the beginning of the experiment and then every 4 days during the 1-month treatment. First, we found that CL-316,243 injections induced persisting weight loss in both NonTg and 3xTg-AD mice (Fig. 1b, c for AUC statistical comparison). At the beginning of the experiment, food consumption recorded in the previous 21 days was higher in 3xTg-AD compared to NonTg mice (average of 4.7 g/day/mice for 3xTg-AD versus 3.9 g/day/mice for NonTg mice; unpaired *t*-test with Welch’s correction: *p* = 0.0105) (Fig. 1d and e for AUC statistical comparison), consistent with lower plasmatic leptin in fasted 3xTg-AD mice compared to NonTg (Fig. 1f). Over the 1-month period, β3AR stimulation increased food consumption in NonTg mice up to levels of transgenic mice (Fig. 1d, e for AUC statistical comparison). The GTT revealed that CL-316,243-treated mice displayed a stronger control over glucose levels compared to control, independently of the genotype (Fig. 1g, h for AUC statistical comparison). Fasting blood glucose and insulin were also lower following 3 weeks of CL-316,243 administration (Fig. 1i, j). However, levels of plasmatic triglycerides were unchanged (Fig. 1k). Overall, our data indicate that β3AR stimulation led to an improved pattern of metabolic determinants in the periphery in both NonTg and 3xTg-AD mice at 15–16 months of age.

### β3AR stimulation increases brown adipose tissue thermogenesis

**Fig. 2:**
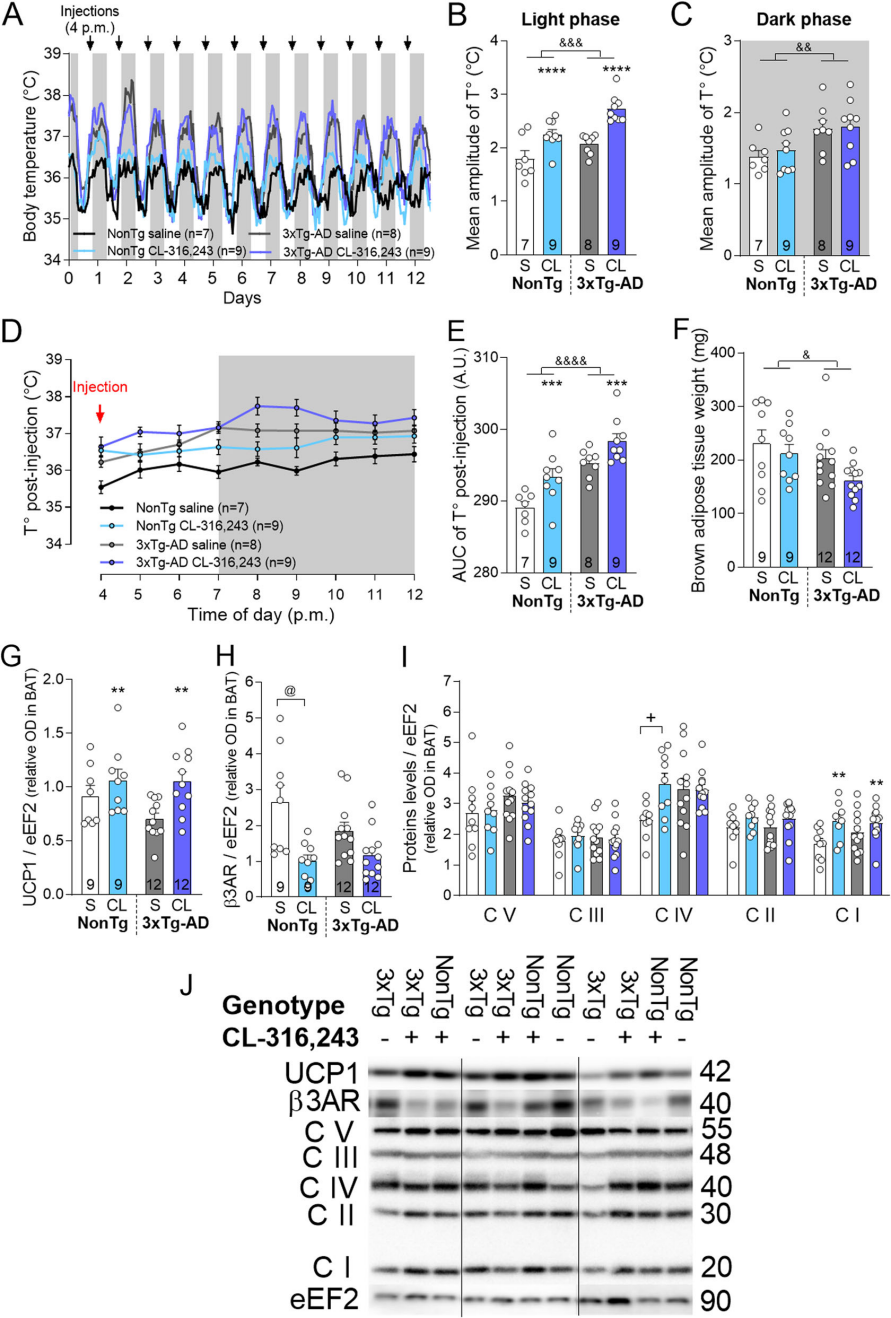
CL-316,243 treatment increases brown adipose tissue thermogenesis. **a** Graphical representation of body temperature recorded hourly by telemetric probe implanted in the intraperitoneal cavity during the first 2 weeks of experiment (before glucose tolerance and behavioral tests). Grey rectangles indicate the dark phase and black arrows point toward each i.p. injection of CL-316,243 or saline. **b** Mean amplitude of body temperature during the light (12-h, from 7 a.m.) and **c** the dark phase (12-h, from 7 p.m.). **d** Body temperature (T°) and **e** area under curve of the T° after the first CL-316,243 or saline i.p. injection. **f** Interscapular BAT weights sampled at sacrifice. Levels of **g** UCP1, **h** β3AR, and **i** mitochondrial oxidative phosphorylation system normalized on eEF2 proteins measured in BAT by Western Blot. **j** Examples of Western blots in BAT samples. Homogenates were all run on the same gel, but consecutive bands were not taken for all representative photo examples. Data are represented as mean ± SEM (*n*/group indicated in each column). Statistics: two-way ANOVA, effect of treatment: **p < 0.01; ***p < 0.001; ****p < 0.0001; effect of genotype: ^&^*p* < 0.05; ^&&^*p* < 0.01; ^&&&^*p* < 0.001; ^&&&&^*p* < 0.0001 (**b**, **c**, **e**–**g**, **i**); Tukey’s post hoc test: ^+^*p* < 0.05. Kruskal-Wallis (**i**), Dunn’s post hoc test: ^@^*p* < 0.05 (**h**). Abbreviations: AUC: area under curve; 3xTg-AD: triple transgenic mice; β3AR: β3 adrenergic receptor; BAT: brown adipose tissue; CL: CL-316,243-injected group; CV, CIII, CIV, CII, CI: mitochondrial oxidative phosphorylation system complex V, III, IV, II, I; eEF2: eukaryotic elongation factor 2; NonTg: non-transgenic mice; OD: optical density; S: saline-injected group; T°: temperature; UCP1: uncoupling protein 1

A telemetric probe implanted a week before the beginning of the treatment revealed daily variation in body temperature corresponding to the sleep-wake cycles of mice (Fig. 2a, Fig. S1). The mean amplitude of body temperature was larger by 0.4 °C in 3xTg-AD than in NonTg mice during both 12-h light and 12-h dark phases (Fig. 2a–c, Fig. S1D). CL-316,243 treatment further increased the amplitude of body temperature during the light phase (from 7 a.m. to 7 p.m.) compared to saline injections (Fig. 2b), corresponding to the injection time, but not during the dark phase (Fig. 2c). This is consistent with higher area under curve of body temperature measured after the first injection of CL-316,243 (between 4 p.m. and 12 p.m.) (Fig. 2d and e for AUC statistical comparison).

Since CL-316,243 is well known to improve thermogenesis capacity in mice [39, 63, 64], we then verified whether it was also effective in old NonTg and 3xTg-AD mice. First, interscapular BAT weight was slightly lower in 3xTg-AD compared to NonTg mice, but was not affected by the treatment (Fig. 2f). However, CL-316,243 administration increased the level of UCP1 protein in the BAT of both genotypes (Fig. 2g) but did not affect the norepinephrine content (Additional File 1). Further confirming that CL-316,243 interacted with β3AR, levels of β3AR in BAT were significantly decreased only in NonTg-treated mice, despite a tendency also in 3xTg-AD mice (Fig. 2h). We then measured complexes I to V of the mitochondrial oxidative phosphorylation complex that are involved in heat production during thermogenesis in BAT [65]. Complex I was increased in NonTg and 3xTg-AD mice following CL-316,243 administration (Fig. 2i, j). CL-316,243 increased complex IV in NonTg, but not in 3xTg-AD mice, whereas complexes II, III, and V remained unchanged in both models. Altogether, our data show that the β3AR agonist administration improves BAT thermogenesis and heat production in 16-month-old mice.

### β3AR stimulation reverses memory deficits in 16-month-old 3xTg-AD mice

**Fig. 3:**
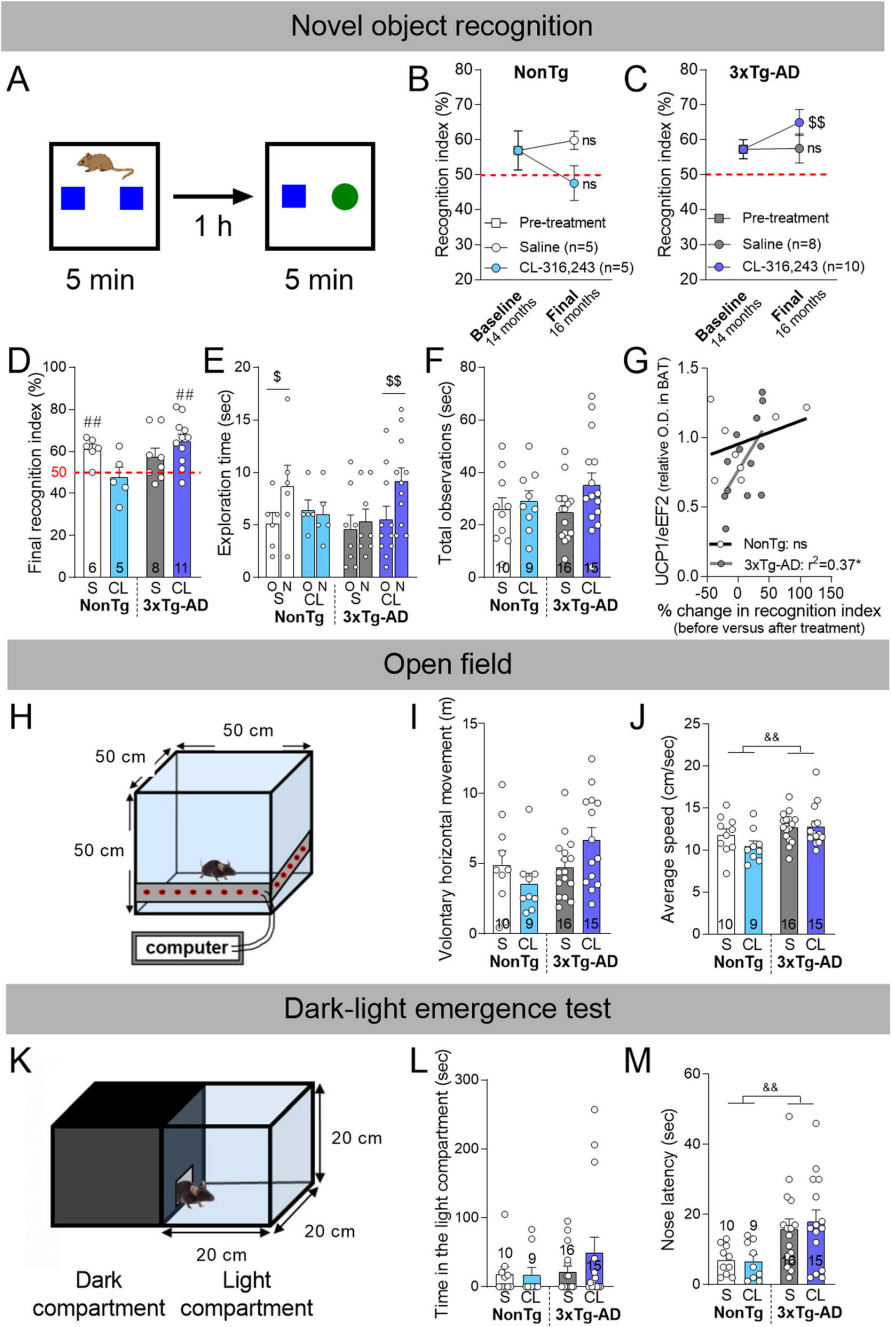
β3AR stimulation reverses memory deficits in 16-month-old 3xTg-AD. **a** Description of the novel object recognition test. Recognition indexes (RI) assessed before (14-month-old mice) and after (16-month-old mice) the treatment with CL-316,243 or saline in **b** NonTg and **c** 3xTg-AD mice. **d** Final recognition index, **e** time spent exploring the old (O), and the novel (N) object during the 5-min acquisition phase and **f** total observations measured at the end of the experiment (final, 16-month-old mice). **g** Correlation between the % change in recognition index (the change in RI before versus after the treatment) and UCP1 levels measured in BAT. **h** Representation of the open field apparatus. **i** Total distance traveled and **j** average speed during the 1-h test. **k** Representation of the dark-light emergence test. **l** Time spent in the light compartment and **m** latency before the first exploration in the light compartment. Recognition index = (time exploring the novel object / total exploration time) × 100. Data are represented as mean ± SEM (*n*/group indicated in graphics). Statistics: Paired *t*-test (baseline versus final recognition index (**b**, **c**); old (O) versus novel (N) object (**e**)): ^$^*p* < 0.05; ^$$^*p* < 0.01 (**b**, **c**, **e**); ns: non-significant compared to recognition index at baseline (**b**, **c**). Dotted red line: 50% RI corresponds to an equal (random) exploration between the novel and the familiar object. One sample *t*-test versus 50% (random chance): ^##^*p* < 0.01 (**d**). Pearson *r* correlation: *p < 0.05 (**g**). Two-way ANOVA, effect of genotype: ^&&^*p* < 0.01 (**j**, **m**). Two-way ANOVA, effect of genotype or treatment: ns (**d**). Abbreviations: 3xTg-AD: triple transgenic mice; CL: CL-316,243-injected group; eEF2: eukaryotic elongation factor 2; NonTg: non-transgenic mice; O.D.: optical density; S: saline-injected group; UCP1: uncoupling protein 1

To determine whether CL-316,243 treatment exerted cognitive benefits in the 3xTg-AD mouse, recognition memory was evaluated with the NOR test 3 weeks before the beginning of the treatment (baseline, 14-month-old) and after the 1-month treatment (final, 16-month-old) (Fig. 1a). The NOR test was selected because of its sensitivity and reliability to detect memory deficits in the 3xTg-AD mice at 12 months and older (Fig. 3a) [9, 56, 57]. Comparing RI before (14 months) and after (16 months) the treatment revealed that 1-month treatment with CL-316,243 increased by 19% the ability to recognize the new object in 3xTg-AD mice (paired *t*-test: *p* = 0.0041), while the change in RI was not significantly different in NonTg or saline-injected 3xTg-AD mice (Fig. 3b, c). CL-316,243 from 15 to 16 months improved memory recognition in 3xTg-AD mice (RI = 65% in CL-316,243-injected mice, one sample *t*-test versus 50%: *p* = 0.0013), but not in NonTg mice (Fig. 3d). The improved RI in 3xTg-AD-treated mice was confirmed by the higher time spent on the novel (N) versus the familial (O) object (Fig. 3e). These differences were not explained by changes in exploratory behavior, as the mean duration of exploration was similar between groups (Fig. 3f). Finally, the percent change in RI before and after the treatment was positively correlated with UCP1 levels in BAT in 3xTg-AD (*r*^2^ = 0.37) but not in NonTg mice, suggesting a link between improved thermogenesis and memory (Fig. 3g).

We then verified that locomotor activity was not affected by CL-316,243 injections, as showed by comparable distance traveled and average speed of the mice during the open field test (Fig. 3h–j). Nonetheless, 2-way ANOVA revealed that 3xTg-AD mice displayed a higher average speed during the 1-h session compared to NonTg mice (Fig. 3j).

Anxiety is frequently observed in AD patients and has been replicated in 3xTg-AD mice [9, 46, 66]. The time spent in the illuminated compartment was not significantly different between groups in the present cohorts of animals (Fig. 3l). However, 3xTg-AD mice delayed their first exploration in the light chamber, as measured by the latency of the first nose entry in the light compartment (nose poke latency) (Fig. 3m), corroborating an anxiety-like behavior in this model.

Overall, 1-month administration of CL-316,243 improved recognition memory assessed at 16 months in 3xTg-AD mice, without affecting locomotion nor anxiety-like behavior.

### β3AR stimulation reduces insoluble Aβ42/Aβ40 ratio in the hippocampus of 3xTg-AD mice

**Fig. 4:**
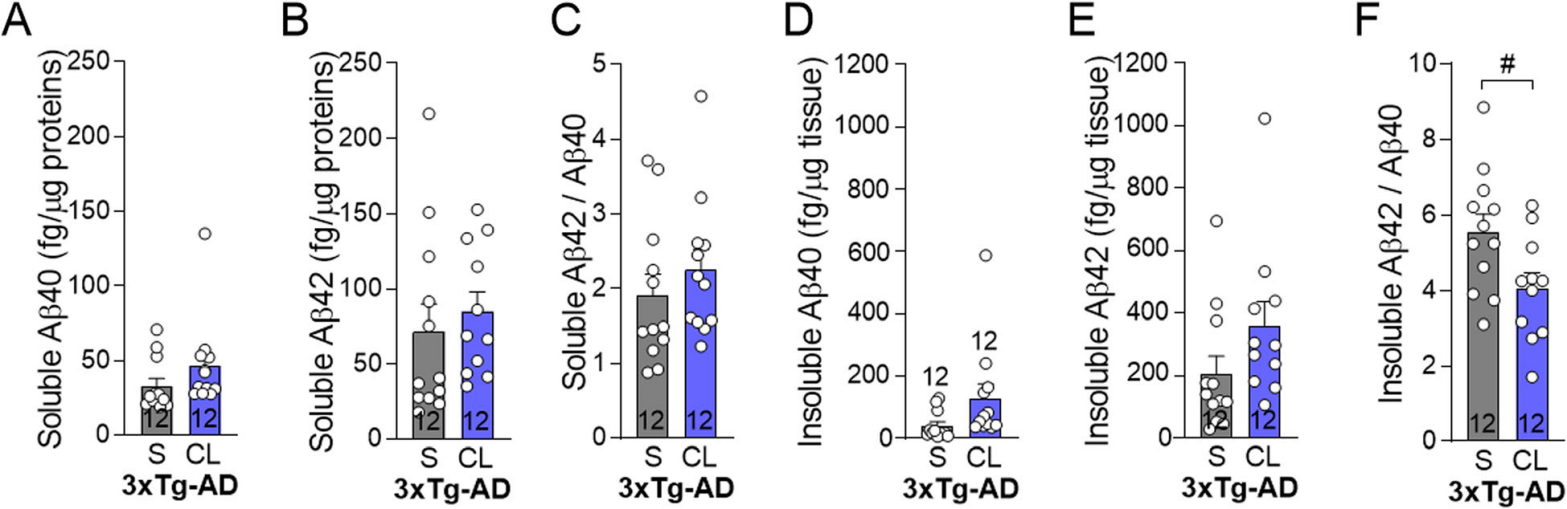
β3AR stimulation reduces insoluble Aβ42/Aβ40 ratio in the hippocampus of 3xTg-AD mice. Human Aβ40 and Aβ42 peptides measured by ELISA in the **a**, **b** detergent-soluble and in the **d**, **e** detergent-insoluble fractions of hippocampus homogenates of 3xTg-AD mice, respectively. **c** Ratio of detergent-soluble and **f** detergent-insoluble Aβ42 on Aβ40 peptides in the hippocampus. Data are represented as mean ± SEM (*n*/group indicated in bars). Statistics: Unpaired Student’s *t* test: ^#^*p* < 0.05 (**a**–**e**). Abbreviations: 3xTg-AD: triple transgenic mice; Aβ: amyloid-β; CL: CL-316,243-injected group; NonTg: non-transgenic mice; S: saline-injected group

The main neuropathological markers of AD, amyloid plaques and tau pathology [67, 68], progressively develop in the brain of 3xTg-AD mice [44, 45, 47]. Although total Aβ42 and Aβ40 peptides in either soluble or insoluble fractions remained unchanged by the treatment in the hippocampus (Fig. 4a–e), we observed a 27% decrease in insoluble Aβ42/Aβ40 ratio in CL-316,243-treated mice compared to saline-injected 3xTg-AD mice (Fig. 4f). We subsequently assessed the effect of the β3AR agonist on proteins implicated in the production (beta-secretase 1 (BACE-1), amyloid precursor protein (APP), APP C-terminal, sAPPα) and clearance or degradation (low density lipoprotein receptor related protein 1 (LRP1), receptor of advanced glycation end products (RAGE), insulin-degrading enzyme (IDE), X11α) of Aβ peptides (Additional File 2) [67, 69, 70]. Levels of BACE-1, IDE, LRP1, and RAGE in the hippocampus did not differ between groups, whereas those of X11α were lower in 3xTg-AD mice compared to NonTg mice (Additional File 2) as observed in the brain of AD individuals [61].

**Fig. 5:**
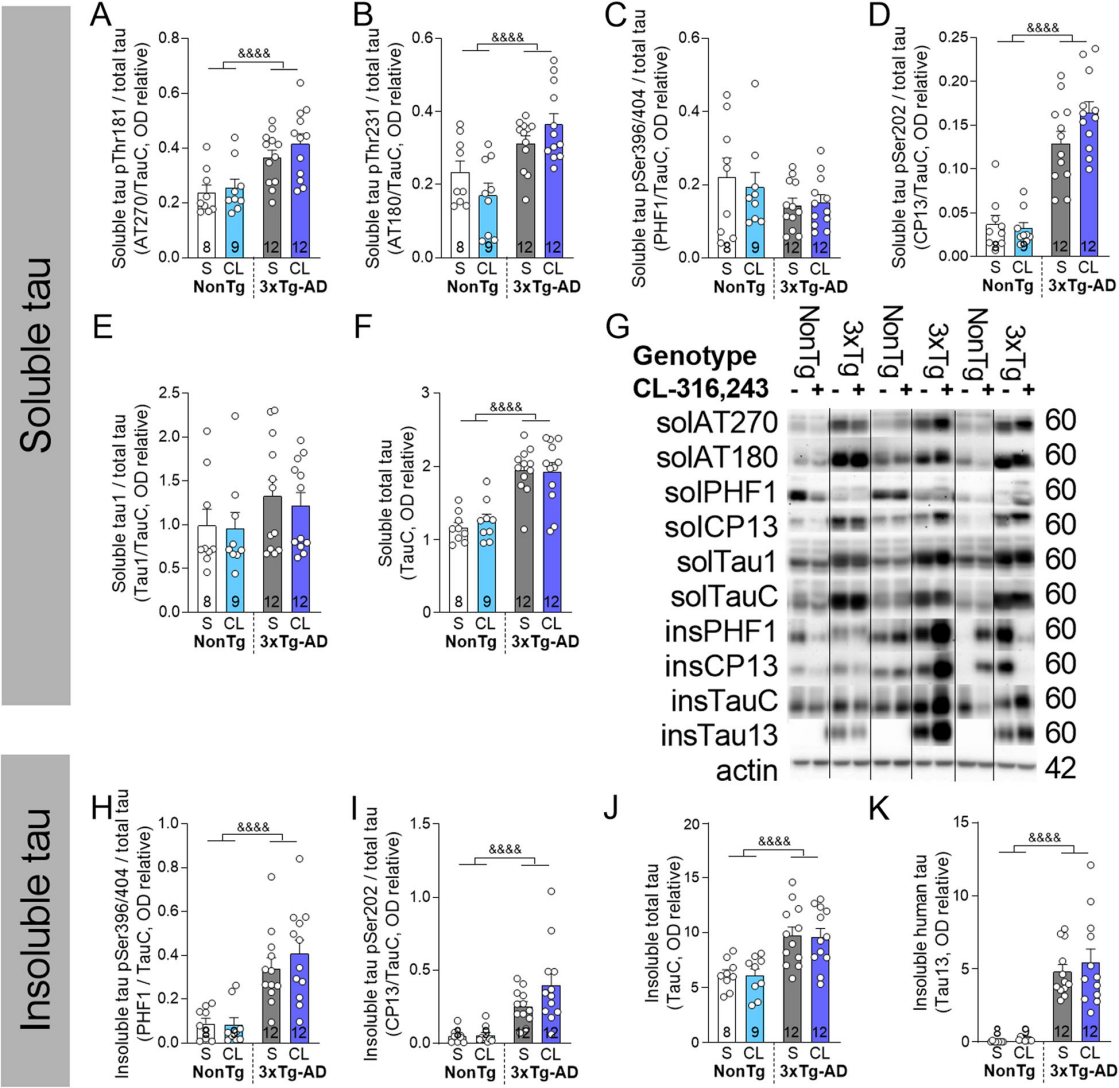
β3AR stimulation has no effect on tau phosphorylation in the hippocampus of 3xTg-AD mice. Phosphorylated and total tau protein measured in **a–f** detergent-soluble and **h**–**k** detergent-insoluble fractions of hippocampus homogenates of non-transgenic and 3xTg-AD mice. **g** Examples of Western blots. Homogenates were all run on the same gel, but consecutive bands were not taken for all representative photo examples and were cut-pasted to match the histogram order. Data are represented as mean ± SEM (*n*/group indicated in bars). Statistics: two-way ANOVA, effect of genotype: ^&&&&^*p* < 0.0001 (**a**–**k**). Abbreviations: 3xTg-AD: triple transgenic mice; CL: CL-316,243-injected group; ins: insoluble; NonTg: non-transgenic mice; OD: optical density; S: saline-injected group; sol: soluble

We then assessed the level of phosphorylated and total tau protein in the detergent-soluble (cytosolic and membrane proteins) and detergent-insoluble (aggregated proteins) fractions of hippocampus homogenates by Western Blot. We did not find any significant effect of CL-316,243 (Fig. 5) but confirmed that the 3xTg-AD mice display higher total and hyperphosphorylated tau proteins compared to NonTg mice. Main kinases involved in tau phosphorylation (glycogen synthase kinase 3 β (GSK3β) and protein kinase B known as AKT) were also unchanged (Additional File 2).

Synaptic deficits are one of the earliest markers of AD, correlating with symptoms [61, 71]. The levels of synaptic proteins were not modified by the treatment (Additional File 2). However, drebrin protein was decreased specifically in 3xTg-AD mice (two-way ANOVA, effect of genotype: *p* = 0.0045), as previously showed in the brain of AD subjects [72, 73].

Since glucose transporters and uptake are decreased in AD [21, 74–76], we assessed glucose transporter 1 (GLUT1) levels in the hippocampus of the mice. While we did not detect any effect of the CL-316,243 treatment, GLUT1 levels were decreased in the hippocampus of 3xTg-AD mice compared to NonTg mice, at both the endothelial (50 kDa) and astrocytic (45 kDa) isoforms (Additional File 2), corroborating defects in glucose uptake, changes in blood-brain barrier transporters, and decreased cerebral vascular volume observed in this mouse model of AD [77–80].

## Discussion

The present study aimed at investigating whether β3AR agonist administration enhances BAT thermogenesis and exerts an effect on cognitive behavior and AD neuropathology in a mouse model of the disease. The 3xTg-AD mice was selected to test the effect of β3AR stimulation in AD because this model displays age-dependent metabolic and thermoregulatory deficits and was shown to respond to thermoneutrality and BAT stimulation induced by repeated cold exposure [8, 9, 16]. This led to the hypothesis that pharmacological BAT stimulation could exert benefits on AD-like behavior and neuropathology.

As a proof of concept, we previously reported that modulating ambient temperature affect AD neuropathology and behavior in mice [9]. It could thus be tempting to deduce that alleviating AD pathogenesis in the elderly can be achieved simply by “turning up the heat.” However, this strategy would probably be inefficient in the long term. Indeed, such as a muscle during exercise, the BAT needs to be trained and prepared to maintain thermogenic capacity continuously [28]. Exposing mammals to a constant thermoneutral environment may induce BAT regression due to no recruitment, letting the organism vulnerable to any further temperature challenge and even inducing subtle hypothermia, leading to aggravated AD neuropathology. Therefore, chronic BAT stimulation, naturally or through a pharmacological intervention, would have the advantage to protect against potential hypothermic events by maintaining an optimal thermogenic capacity.

To test the hypothesis in an animal model of AD, we thus treated 15-month-old NonTg and 3xTg-AD mice with the selective β3AR agonist CL-316,243 or saline for a month. We found that chronic administration of the agonist stimulated BAT thermogenesis and improved glucose homeostasis. Enhanced thermogenesis was associated with improved recognition memory in 16 months 3xTg-AD mice and reduced insoluble Aβ42/Aβ40 ratio in the hippocampus, while tau pathology remained unaffected.

### β3AR agonist: a two in one strategy to target both metabolic and thermoregulatory defects

The most well-known characteristic of CL-316,243 is to improve metabolic disorders through enhanced BAT activity [36–40]. Whether this effect is maintained with age remains unknown. UCP1 and NADH dehydrogenase 1 beta subcomplex subunit 8 (NDUFB8 or Complex I) are both recognized markers of BAT activation (Nam and Cooper, 2015). Higher levels of UCP1 and Complex I in the BAT detected here indicate a sustained effect of CL-316,243 on BAT thermogenesis after a 1-month chronic treatment in old mice. This is consistent with strong previous evidence that β3AR stimulation leads to increased thermogenic activity of BAT in young mice (Labbé et al., 2016; Poher et al., 2015; Xiao et al., 2015) and humans as well [41, 81]. It also shows that chronic β3AR administration does not induce significant receptor desensitization, despite decreased levels of BAT β3AR following treatment, in agreement with previous works [82]. Overall, our data now confirms that β3AR-induced thermogenesis is still effective in a 15-month-old mouse, following a 1-month treatment with CL-316,243, regardless of the presence of AD neuropathology.

White adipose tissue “beiging” (i.e., white adipocyte expressing UCP1) is another marker of enhanced thermogenesis in rodents [83] which could be investigated after CL-316,243 treatment. However, compared to BAT activity, “beige” cell contribution to thermogenesis is negligible [39]. Nevertheless, in the present study, β3AR agonist induced weight loss in both NonTg and 3xTg-AD mice, suggesting increased lipolysis. Food consumption was higher in 3xTg-AD compared to NonTg mice, an observation previously reported in this model [84, 85], and CL-316,243 increased food intake in NonTg mice. These data are consistent with increased energetic expenditure compensated with higher calorie intake following β3AR stimulation [64, 86].

We previously showed that female 3xTg-AD mice display age-dependent glucose intolerance starting from 12 months [47]. While the difference in glucose tolerance between NonTg and 3xTg-AD mice was not frank in the present work, probably due to the fact that only males were used, fasting blood glucose in 3xTg-AD was higher than in NonTg mice. It is noteworthy that the CL-316,243 improved peripheral glucose metabolism and fasted insulin in 16-month-old mice of both genotypes, suggesting an effect independent of AD neuropathology. Of note, the improvement was observed in mice fed with a diet not expected to induce metabolic defects, suggesting benefits even in non-diabetic animals. This is in line with a previous study showing improved glucose metabolism with CL-316,243 administration even in mice displaying a normal response to glucose [64]. Thus, a β3AR agonist treatment could also benefit elderly individuals without diabetes.

Using hourly telemetric recordings of body temperature, we noted that 3xTg-AD mice displayed wider amplitudes of body temperature throughout the day compared to NonTg mice. On the other hand, β3AR agonist treatment increased amplitude of body temperature during the light phase, corresponding to the period of drug administration, but not during the dark phase. The wider amplitude in body temperature is consistent with a higher body temperature after CL-316,243 injection, as shown by the temperature recorded few hours after the first i.p. injection and a previous work [87]. However, we did not observed chronic hyperthermia, which would have been a major side effect of β3AR agonists, perhaps compromising potential translation to clinical use.

### β3AR stimulation reverses memory deficits in old 3xTg-AD mice

An important result of our study is the reversal of recognition memory deficit induced by β3AR stimulation in 16-month-old 3xTg-AD mice. Indeed, we observed a 19% increase of RI between baseline (14 months) and post-treatment evaluation (16 months). These results were not explained by changes in exploratory behavior or locomotor activity. The RI of NonTg mice following treatment did not reach statistical significance perhaps due to lower statistical power (*n* = 5), but was significantly different from 50% in saline-injected mice. Although a downward trend was noted, the RI of the NonTg group treated with CL-316,243 was not significantly different after versus before the treatment. However, the high interindividual variability and the low number of mice in the group prevent us to draw a conclusion concerning potential positive or negative effects in NonTg mice. Reassuringly, a recent study shows no negative effect on the memory performance after a 3-month treatment with mirabegron in patients with overactive bladder aged ≥ 65 years [88]. Nevertheless, the data also suggest that β3AR-induced improvement in memory was specific to 3xTg-AD mice, possibly through an effect related to AD neuropathology. As only 3xTg-AD mice develop Aβ plaques or neurofibrillary tangles, if CL-316,243 exerted an AD-relevant effect on Aβ and tau pathologies, it would be detected only in 3xTg-AD mice. This may explain the present difference in response between NonTg and 3xTg-AD mice. Indeed, regarding the effects of CL-316,243 administration on classical AD neuropathology, we observed a reduction in the Aβ42:Aβ40 ratio within insoluble deposits, which is only quantifiable in transgenic mice. Importantly, the randomized-start design ensured that all groups of animals were similar before undergoing saline or CL-316,243 treatment. One study also found memory improvement following CL-316,243 administration in Aβ-injected chicks [89], but no previous report in mice was found in the literature. Thus, our results are consistent with a disease-modifying effect of β3AR stimulation in the 3xTg-AD mice.

Since metabolic disorders also alter cognitive function and lead to memory defects [90–94], improved peripheral metabolism could be involved in better recognition memory in 3xTg-AD mice. However, it is not excluded that CL-316,243 has a direct effect in the CNS. Indeed, β3AR are present in various regions of the brain, although to a much lower extent compared to the BAT [95, 96], but their physiological roles in the central nervous system (CNS) are not known. While it has been shown that CL-316,243 increases sleep duration in mice [87] and reduces Aβ-induced long-term memory deficits in chicks [89], the behavioral effects of β3AR agonists has not been the subject of intense investigation. Nonetheless, another β3AR agonist, SR856611A (Amibegron®), has been shown to improve anxiety and depressive-like symptoms in rodents, as evaluated by the forced swim test and the elevated plus maze [97–99]. This promising molecule reached phase III clinical trial for depression (NCT00252330) but did not achieve final approval to move into the clinic. In the present work, we did not detect changes in anxiety-like behavior with the dark-light emergence test following CL-316,243 administration. Recently, a group reported no change in MoCA score in a group of elderly receiving mirabegron (Myrbetriq®) as a treatment for overactive bladder for 3 months [88]. However, only 115 volunteers with impaired cognitive performance received the treatment, and 3 months might be too short to detect any potential cognitive benefits [100]. While the effects of β3AR agonists on the brain are not yet clearly understood, what stand up from our data is that enhanced UCP1 levels in BAT were correlated with higher improvement in RI in 3xTg-AD mice, supporting the idea that higher BAT thermogenesis induced by β3AR stimulation is involved in improved memory performance.

### β3AR stimulation decreases insoluble Aβ42/Aβ40 ratio but has no effect on tau phosphorylation in the hippocampus of 3xTg-AD mice

The 3xTg-AD model allowed us to probe whether the effect of CL-316,243 are related to changes in AD neuropathology. Despite no change in total tau or Aβ burden, a 27% decrease in insoluble Aβ42/Aβ40 ratio was observed in the hippocampus of old 3xTg-AD mice following CL-316,243 injections. We did not observe concomitant changes in Aβ40, Aβ42, BACE1, APP, IDE, or LRP1. Although no clear mechanism can be identified from our study, genetic data strongly suggest that a treatment reducing the Aβ42/Aβ40 ratio in the hippocampus is consistent with a beneficial impact on Aβ production and aggregation [101, 102]. Aβ42/Aβ40 ratio in the brain has been consistently associated with higher risk of developing AD, at least in genetic cases. Indeed, the Aβ42/Aβ40 ratio is increased in familial forms of AD and is inversely correlated with the age of onset of the disease [103, 104]. Decreased Aβ42/Aβ40 ratio suggests a shift in APP cleavage from Aβ42 to Aβ40, which is less prone to aggregation than Aβ42. Importantly, increased Aβ42/Aβ40 ratio precedes amyloid plaques formation in the Tg2576 mouse model of AD [105]. More recent work using induced pluripotent stem cells (iPSC) corroborates this view, indicating that APP or presenilin 1 (PSEN1) mutations, the more likely to cause familial AD, also leads to higher Aβ42/Aβ40 ratio [106]. Consistent with biochemical data in cellular models, recent clinical studies suggest that CSF and plasma Aβ ratios are biomarkers of Aβ processing and can be useful in the diagnosis of AD [101, 107].

The absence of changes on the phosphorylation status of tau following CL-316,243 administration could be interpreted as surprising. Since tau phosphorylation has been repeatedly shown to follow body temperature modulation [12–14], we could have expected a protective effect of β3AR stimulation. We observed a transitory increase in temperature following CL-316,243 injection, but this effect was only temporary and thus did not impact tau phosphorylation measured at the end of the experiment. As mentioned above, CL-316,243 did not induce chronic hyperthermia, which would actually be an adverse effect. In contrast, we recently showed that improved BAT thermogenesis through repeated cold exposure protects old 3xTg-AD mice from cold-induced tau phosphorylation [16]. However, the present study design did not fully explore the hypothesis that β3AR stimulation impacts tau phosphorylation, because the animals were kept at room temperature and not exposed to any frank thermoregulatory challenge (i.e., acute 24-h exposure to cold). Thus, because it has not been directly tested in the present study, it remains possible that pharmacological β3AR stimulation also confers protection against cold-induced tau phosphorylation.

### Limitations

Limitations of the current work include the absence of confirmation that CL-316,243 crosses the blood-brain barrier and had a direct effect in the CNS of mice. However, as stated above, reaching the brain is not absolutely required to exert benefits, as the prime target of β3AR agonists is the BAT. Another limitation is the low number of mice in the NonTg group that met the inclusion criteria of minimal exploring time to be included in the NOR test, preventing us to draw conclusion on a potential effect of β3AR agonist on old control (NonTg) mice. However, our several previous studies showed that this memory test is appropriate and adapted for old 3xTg-AD mice, because it is less affected by other variables failing with age in these types of models, such as thermoregulation, sensorimotor performance, and anxiety. Furthermore, the effect of a β3AR agonist was evaluated in only one mouse model of AD, the 3xTg-AD mouse model of AD. It is important to note that there is no true mouse model of sporadic AD available. Although the triple transgenic mice is a model generated by the overexpression of three human mutations found in familial forms of AD, it is the only one that recapitulates both progressive tau and Aβ pathologies as well as thermoregulatory and metabolic impairments [9, 44]. However, it would be interesting to investigate the effect of a β3AR agonist in other recently developed murine models of tau or Aβ pathologies [108]. Finally, we used only males to avoid temperature variations induced by the estrous cycle of female mice, but since female 3xTg-AD mice develop aggravated metabolic deficits as well as higher Aβ pathology compared to males, the benefit of the treatment observed could have been more prominent in females.

## Conclusions

The present work aimed to determine whether β3AR agonists exert positive effects on AD-relevant endpoints in an animal model of tau and Aβ neuropathology, thereby providing arguments for drug repurposing in AD. This class of drugs is actively being tested in clinical studies for metabolic diseases [109, 110] and CL-316,243 has been previously investigated in humans as well [111]. As an example, mirabegron (Myrbetriq®) has been approved by the Food and Drug Administration for the treatment of overactive bladder [41, 112], and large randomized controlled trials have confirmed its safety and tolerability profiles [113], even in the elderly population [114]. Thus, the potential translation to clinical use of this class of drugs in AD is high. Nonetheless, it has to be noted that in humans, potential side effects of β3AR agonists include cardiovascular dysfunction induced by non-specific activation of β1 and β2 adrenergic receptors. Indeed, a single administration of mirabegron at a dose of 200 mg (fourth times the clinical dose) enhances BAT activity in adults, but also induced cardiac arrhythmia in a few cases [41]. While the clinical dose of 50 mg does not seem to be efficient to acutely stimulate BAT [42], a recent study showed that 100 mg of mirabegron enhances thermogenesis without any cardiovascular side effects in adults [81]. Yet, long-term studies investigating chronic effect of β3AR agonists in BAT thermogenesis in old volunteers are needed.

Altogether, our results in a mouse model of AD demonstrate for the first time that β3AR agonists are potent tools to reverse memory deficits and insoluble Aβ42/Aβ40 ratio in the hippocampus. It is the first study to our knowledge to investigate the potential of this class of drugs on AD neuropathology and behavior.

## Supplementary Information

**Additional file 1. **CL-316,243 administration does not affect circadian rhythm parameters nor BAT norepinephrine content. A: individual mean daily offsets (determined as the time of the first six successive bins when temperature was lower than the mean diurnal temperature, thus corresponding to morning temperature drop). B: Offset standard deviation. C: Mean duration of a total temperature cycle. D: Mean amplitude of body temperature during one day (24-h, from 7 a.m. to 7 p.m.). E: Norepinephrine concentrations measured by HPLC in BAT, normalized to tissue weights. Data are represented as mean ± SEM (n/group indicated in bars). Statistics: Two-way ANOVA, effect of CL-316,243 treatment: ****p < 0.0001, effect of genotype: ^&^*p* < 0.05 ^&&&&^*p* < 0.0001 (A-E). Abbreviations: 3xTg-AD: triple transgenic mice; CL: CL-316,243-injected group; NonTg: non-transgenic mice; S: saline-injected group

**Additional file 2. **Other AD markers not affected by CL-316,243 treatment. Relative optical density of proteins normalized on actin measured in detergent-soluble fraction of hippocampus homogenates by Western Blot. Data are represented as mean ± SD, *n* = 9-12 per group. Abbreviations: AKT: protein kinase B; APP: amyloid precursor protein; BACE-1: beta-secretase 1; GFAP: glial fibrillary acidic protein; GLUT1: glucose transporter 1; GSK3β, glycogen synthase kinase 3β; IDE: insulin degrading enzyme; LRP1: low density lipoprotein receptor related protein 1; PSD95: post-synaptic density 95; RAGE: receptor of advanced glycation end products; sAPPα: soluble α-APP

**Additional file 3.** Antibodies used in this study

## Data Availability

The datasets generated and analyzed during the current study are available from the corresponding author on reasonable request.

## Abbreviations

3xTg-AD: Triple transgenic mouse model of AD
β3AR: β3 adrenergic receptor
Aβ: Amyloid-β
AD: Alzheimer’s disease
AKT: Protein kinase B
APP: Amyloid precursor protein
AUC: Area under curve
BACE-1: Beta-secretase 1
BAT: Brown adipose tissue
CV, CIII, CIV, CII, CI: Mitochondrial oxidative phosphorylation system complex V, III, IV, II, I
DL: Dark-light emergence test
eEF2: Eukaryotic elongation factor 2
GFAP: Glial fibrillary acidic protein
GLUT1: Glucose transporter 1
GSK3β: Glycogen synthase kinase 3 β
GTT: Glucose tolerance test
IDE: Insulin degrading enzyme
LRP1: Low-density lipoprotein receptor related protein 1
NonTg: Non-transgenic mice
NOR: Novel object recognition test
OD: Optical density
OF: Open field test
PSD95: Post-synaptic density 95
PSEN1: Presenilin 1
RAGE: Receptor of advanced glycation end products
RI: Recognition index
sAPPα: soluble α-APP
T°: Temperature
T2D: Type 2 diabetes
UCP1: Uncoupling protein 1

## References

[1] Scheltens P, Blennow K, Breteler MMB, De Strooper B, Frisoni GB, Salloway S (2016). Alzheimer’s disease. Lancet..

[2] Querfurth HW, LaFerla FM (2010). Alzheimer’s disease. N Engl J Med.

[3] Winblad B, Amouyel P, Andrieu S, Ballard C, Brayne C, Brodaty H, Cedazo-Minguez A, Dubois B, Edvardsson D, Feldman H, Fratiglioni L, Frisoni GB, Gauthier S, Georges J, Graff C, Iqbal K, Jessen F, Johansson G, Jönsson L, Kivipelto M, Knapp M, Mangialasche F, Melis R, Nordberg A, Rikkert MO, Qiu C, Sakmar TP, Scheltens P, Schneider LS, Sperling R, Tjernberg LO, Waldemar G, Wimo A, Zetterberg H (2016). Defeating Alzheimer’s disease and other dementias: a priority for European science and society. Lancet Neurol.

[4] Degroot DW, Kenney WL (2007). Impaired defense of core temperature in aged humans during mild cold stress. Am J Physiol Regul Integr Comp Physiol.

[5] Grassi G, Seravalle G, Turri C, Bertinieri G, Dell'Oro R, Mancia G (2003). Impairment of thermoregulatory control of skin sympathetic nerve traffic in the elderly. Circulation.

[6] Gomolin IH, Aung MM, Wolf-Klein G, Auerbach C (2005). Older is colder: temperature range and variation in older people. J Am Geriatr Soc.

[7] Hebert LE, Weuve J, Scherr PA, Evans DA (2013). Alzheimer disease in the United States (2010-2050) estimated using the 2010 census. Neurology..

[8] Knight EM, Brown TM, Gümüsgöz S, Smith JCM, Waters EJ, Allan SM (2013). Age-related changes in core body temperature and activity in triple-transgenic Alzheimer’s disease (3xTgAD) mice. Dis Model Mech.

[9] Vandal M, White PJ, Tournissac M, Tremblay C, St-Amour I, Drouin-Ouellet J, Bousquet M, Traversy MT, Planel E, Marette A, Calon F (2016). Impaired thermoregulation and beneficial effects of thermoneutrality in the 3 × Tg-AD model of Alzheimer’s disease. Neurobiol Aging.

[10] Sterniczuk R, Dyck RH, LaFerla FM, Antle MC (2010). Characterization of the 3xTg-AD mouse model of Alzheimer’s disease: part 1. Circadian changes. Brain Res.

[11] Huitrón-Reséndiz S, Sánchez-Alavez M, Gallegos R, Berg G, Crawford E, Giacchino JL, Games D, Henriksen SJ, Criado JR (2002). Age-independent and age-related deficits in visuospatial learning, sleep-wake states, thermoregulation and motor activity in PDAPP mice. Brain Res.

[12] Planel E, Richter KEG, Nolan CE, Finley JE, Liu L, Wen Y, Krishnamurthy P, Herman M, Wang L, Schachter JB, Nelson RB, Lau LF, Duff KE (2007). Anesthesia leads to tau hyperphosphorylation through inhibition of phosphatase activity by hypothermia. J Neurosci.

[13] Arendt T, Stieler J, Strijkstra AM, Hut RA, Rüdiger J, Van der Zee EA (2003). Reversible paired helical filament-like phosphorylation of tau is an adaptive process associated with neuronal plasticity in hibernating animals. J Neurosci.

[14] Stieler JT, Bullmann T, Kohl F, Tøien Ø, Brückner MK, Härtig W (2011). The physiological link between metabolic rate depression and tau phosphorylation in mammalian hibernation. PLoS One.

[15] Tournissac M, Vandal M, Francois A, Planel E, Calon F (2017). Old age potentiates cold-induced tau phosphorylation: linking thermoregulatory deficit with Alzheimer’s disease. Neurobiol Aging.

[16] Tournissac M, Bourassa P, Martinez-Cano RD, Vu T-M, Hébert SS, Planel E, Calon F (2019). Repeated cold exposures protect a mouse model of Alzheimer’s disease against cold-induced tau phosphorylation. Mol Metab.

[17] Vandal M, White PJ, Tremblay C, St-Amour I, Chevrier G, Emond V (2014). Insulin reverses the high-fat diet-induced increase in brain aβ and improves memory in an animal model of Alzheimer disease. Diabetes.

[18] Gratuze M, Julien J, Petry FR, Morin F, Planel E (2017). Insulin deprivation induces PP2A inhibition and tau hyperphosphorylation in hTau mice, a model of Alzheimer’s disease-like tau pathology. Sci Rep.

[19] Julien C, Tremblay C, Phivilay A, Berthiaume L, Emond V, Julien P (2010). High-fat diet aggravates amyloid-beta and tau pathologies in the 3xTg-AD mouse model. Neurobiol Aging.

[20] Thériault P, ElAli A, Rivest S. High fat diet exacerbates Alzheimer's disease-related pathology in APPswe/PS1 mice. Oncotarget. 2016;7(42):67808–27. 10.18632/oncotarget.12179. 10.18632/oncotarget.12179 PMC5356521 27661129

[21] An Y, Varma VR, Varma S, Casanova R, Dammer E, Pletnikova O, Chia CW, Egan JM, Ferrucci L, Troncoso J, Levey AI, Lah J, Seyfried NT, Legido-Quigley C, O'Brien R, Thambisetty M (2018). Evidence for brain glucose dysregulation in Alzheimer’s disease. Alzheimers Dement.

[22] Arnold SE, Arvanitakis Z, Macauley-Rambach SL, Koenig AM, Wang H-Y, Ahima RS, Craft S, Gandy S, Buettner C, Stoeckel LE, Holtzman DM, Nathan DM (2018). Brain insulin resistance in type 2 diabetes and Alzheimer disease: concepts and conundrums. Nat Rev Neurol.

[23] Biessels GJ, Staekenborg S, Brunner E, Brayne C, Scheltens P (2006). Risk of dementia in diabetes mellitus: a systematic review. Lancet Neurol.

[24] Yarchoan M, Arnold SE (2014). Repurposing diabetes drugs for brain insulin resistance in Alzheimer disease. Diabetes.

[25] Craft S, Baker LD, Montine TJ, Minoshima S, Watson GS, Claxton A, Arbuckle M, Callaghan M, Tsai E, Plymate SR, Green PS, Leverenz J, Cross D, Gerton B (2012). Intranasal insulin therapy for Alzheimer disease and amnestic mild cognitive impairment: a pilot clinical trial. Arch Neurol.

[26] Liu J, Wang L-N, Jia J-P (2015). Peroxisome proliferator-activated receptor-gamma agonists for Alzheimer’s disease and amnestic mild cognitive impairment: a systematic review and meta-analysis. Drugs Aging.

[27] Gejl M, Gjedde A, Egefjord L, Møller A, Hansen SB, Vang K (2016). In Alzheimer’s disease, 6-month treatment with GLP-1 analog prevents decline of brain glucose metabolism: randomized, placebo-controlled, double-blind clinical trial. Front Aging Neurosci. Frontiers.

[28] Cannon B, Nedergaard J (2004). Brown adipose tissue: function and physiological significance. Physiol Rev.

[29] Virtanen KA, Lidell ME, Orava J, Heglind M, Westergren R, Niemi T, Taittonen M, Laine J, Savisto NJ, Enerbäck S, Nuutila P (2009). Functional brown adipose tissue in healthy adults. N Engl J Med.

[30] Cypess AM, Lehman S, Williams G, Tal I, Rodman D, Goldfine AB, Kuo FC, Palmer EL, Tseng YH, Doria A, Kolodny GM, Kahn CR (2009). Identification and importance of brown adipose tissue in adult humans. N Engl J Med.

[31] Hanssen MJW, Hoeks J, Brans B, van der Lans AAJJ, Schaart G, van den Driessche JJ, Jörgensen JA, Boekschoten MV, Hesselink MKC, Havekes B, Kersten S, Mottaghy FM, van Marken Lichtenbelt WD, Schrauwen P (2015). Short-term cold acclimation improves insulin sensitivity in patients with type 2 diabetes mellitus. Nat Med.

[32] Ravussin Y, Xiao C, Gavrilova O, Reitman ML (2014). Effect of intermittent cold exposure on brown fat activation, obesity, and energy homeostasis in mice. Public Libr Sci.

[33] Schrauwen P, van Marken Lichtenbelt WD (2016). Combatting type 2 diabetes by turning up the heat. Diabetologia.

[34] Arch JRS (2011). Challenges in β(3)-adrenoceptor agonist drug development. Ther Adv Endocrinol Metab.

[35] Nedergaard J, Golozoubova V, Matthias A, Asadi A, Jacobsson A, Cannon B (2001). UCP1: the only protein able to mediate adaptive non-shivering thermogenesis and metabolic inefficiency. Biochim Biophys Acta.

[36] de Souza CJ, Hirshman MF, Horton ES (1997). CL-316,243, a beta3-specific adrenoceptor agonist, enhances insulin-stimulated glucose disposal in nonobese rats. Diabetes..

[37] Burkey BF, Dong M, Gagen K, Eckhardt M, Dragonas N, Chen W, Grosenstein P, Argentieri G, de Souza CJ (2000). Effects of pioglitazone on promoting energy storage, not expenditure, in brown adipose tissue of obese fa/fa Zucker rats: comparison to CL 316,243. Metabolism..

[38] Kumar A, Shiloach J, Betenbaugh MJ, Gallagher EJ (2015). The beta-3 adrenergic agonist (CL-316,243) restores the expression of down-regulated fatty acid oxidation genes in type 2 diabetic mice. Nutr Metab (Lond).

[39] Labbé SM, Caron A, Chechi K, Laplante M, Lecomte R, Richard D (2016). Metabolic activity of brown, “beige,” and white adipose tissues in response to chronic adrenergic stimulation in male mice. Am J Physiol Endocrinol Metab.

[40] Kim H, Pennisi PA, Gavrilova O, Pack S, Jou W, Setser-Portas J (2006). Effect of adipocyte beta3-adrenergic receptor activation on the type 2 diabetic MKR mice. Am J Physiol Endocrinol Metab.

[41] Cypess AM, Weiner LS, Roberts-Toler C, Franquet Elía E, Kessler SH, Kahn PA (2015). Activation of human brown adipose tissue by a β3-adrenergic receptor agonist. Cell Metab.

[42] Baskin AS, Linderman JD, Brychta RJ, McGehee S, Anflick-Chames E, Cero C, et al. Regulation of human adipose tissue activation, gallbladder size, and bile acid metabolism by a β3-adrenergic receptor agonist. Diabetes. 2018. 10.2337/db18-0462 American Diabetes Association. 10.2337/db18-0462 PMC6152342 29980535

[43] Chapple CR, Cardozo L, Nitti VW, Siddiqui E, Michel MC (2014). Mirabegron in overactive bladder: a review of efficacy, safety, and tolerability. Neurourol Urodyn.

[44] Oddo S, Caccamo A, Shepherd JD, Murphy MP, Golde TE, Kayed R, Metherate R, Mattson MP, Akbari Y, LaFerla FM (2003). Triple-transgenic model of Alzheimer’s disease with plaques and tangles. Neuron..

[45] Belfiore R, Rodin A, Ferreira E, Velazquez R, Branca C, Caccamo A (2018). Temporal and regional progression of Alzheimer’ disease-like pathology in 3xTg-AD mice. Aging Cell.

[46] St-Amour I, Paré I, Tremblay C, Coulombe K, Bazin R, Calon F (2014). IVIg protects the 3xTg-AD mouse model of Alzheimer’s disease from memory deficit and Aβ pathology. J Neuroinflammation.

[47] Vandal M, White PJ, Chevrier G, Tremblay C, St-Amour I, Planel E (2015). Age-dependent impairment of glucose tolerance in the 3xTg-AD mouse model of Alzheimer’ disease. FASEB J.

[48] Bories C, Guitton MJ, Julien C, Tremblay C, Vandal M, Msaid M (2012). Sex-dependent alterations in social behaviour and cortical synaptic activity coincide at different ages in a model of Alzheimer’s disease. Public Libr Sci.

[49] Weinert D, Waterhouse J, Nevill A (2004). Changes of body temperature and thermoregulation in the course of the ovarian cycle in laboratory mice. Biol Rhythm Res.

[50] Danysz W, Han Y, Li F, Nicoll J, Buch P, Hengl T, Ruitenberg M, Parsons C (2018). Browning of white adipose tissue induced by the ß3 agonist CL-316,243 after local and systemic treatment - PK-PD relationship. Biochim Biophys Acta.

[51] Ghorbani M, Shafiee Ardestani M, Gigloo SH, Cohan RA, Inanlou DN, Ghorbani P (2012). Anti diabetic effect of CL 316,243 (a β3-adrenergic agonist) by down regulation of tumour necrosis factor (TNF-α) expression. Public Libr Sci.

[52] Bloom JD, Dutia MD, Johnson BD, Wissner A, Burns MG, Largis EE, Dolan JA, Claus TH (1992). Disodium (R,R)-5-[2-[[2-(3-chlorophenyl)-2-hydroxyethyl]-amino] propyl]-1,3-benzodioxole-2,2-dicarboxylate (CL 316,243). A potent beta-adrenergic agonist virtually specific for beta 3 receptors. A promising antidiabetic and antiobesity agent. J Med Chem.

[53] Yoshida T, Sakane N, Wakabayashi Y, Umekawa T, Kondo M (1994). Anti-obesity and anti-diabetic effects of CL 316,243, a highly specific beta 3-adrenoceptor agonist, in yellow KK mice. Life Sci.

[54] Caron A, Labbé SM, Carter S, Roy M-C, Lecomte R, Ricquier D, Picard F, Richard D (2017). Loss of UCP2 impairs cold-induced non-shivering thermogenesis by promoting a shift toward glucose utilization in brown adipose tissue. Biochimie..

[55] Dal-Pan A, Dudonné S, Bourassa P, Bourdoulous M, Tremblay C, Desjardins Y (2016). Cognitive-enhancing effects of a polyphenols-rich extract from fruits without changes in neuropathology in an animal model of Alzheimer’s disease. J Alzheimers Dis.

[56] Clinton LK, Billings LM, Green KN, Caccamo A, Ngo J, Oddo S, McGaugh JL, LaFerla FM (2007). Age-dependent sexual dimorphism in cognition and stress response in the 3xTg-AD mice. Neurobiol Dis.

[57] Arsenault D, Julien C, Tremblay C, Calon F (2011). DHA improves cognition and prevents dysfunction of entorhinal cortex neurons in 3xTg-AD mice. PLoS One.

[58] Wolf A, Bauer B, Abner EL, Ashkenazy-Frolinger T, Hartz AMS (2016). A Comprehensive behavioral test battery to assess learning and memory in 129S6/Tg2576 mice. PLoS One.

[59] Leger M, Quiedeville A, Bouet V, Haelewyn B, Boulouard M, Schumann-Bard P, Freret T (2013). Object recognition test in mice. Nat Protoc.

[60] Antunes M, Biala G (2012). The novel object recognition memory: neurobiology, test procedure, and its modifications. Cogn Process.

[61] Tremblay C, Francois A, Delay C, Freland L, Vandal M, Bennett (2017). Association of neuropathological markers in the parietal cortex with antemortem cognitive function in persons with mild cognitive impairment and Alzheimer disease. J Neuropathol Exp Neurol.

[62] Bousquet M, Gue K, Emond V, Julien P, Kang JX, Cicchetti F, Calon F (2011). Transgenic conversion of omega-6 into omega-3 fatty acids in a mouse model of Parkinson's disease. J Lipid Res.

[63] Poher A-L, Veyrat-Durebex C, Altirriba J, Montet X, Colin DJ, Caillon A, Lyautey J, Rohner-Jeanrenaud F (2015). Ectopic UCP1 overexpression in white adipose tissue improves insulin sensitivity in Lou/C rats, a model of obesity resistance. Diabetes..

[64] Xiao C, Goldgof M, Gavrilova O, Reitman ML (2015). Anti-obesity and metabolic efficacy of the β3-adrenergic agonist, CL316243, in mice at thermoneutrality compared to 22 °C. Obesity (Silver Spring).

[65] Nam M, Cooper MP (2015). Role of energy metabolism in the brown fat gene program. Front Endocrinol (Lausanne).

[66] Hebda-Bauer EK, Simmons TA, Sugg A, Ural E, Stewart JA, Beals JL (2013). 3xTg-AD mice exhibit an activated central stress axis during early-stage pathology. J Alzheimers Dis.

[67] Selkoe DJ, Hardy J (2016). The amyloid hypothesis of Alzheimer’ disease at 25 years. EMBO Mol Med.

[68] Iqbal K, Liu F, Gong C-X (2016). Tau and neurodegenerative disease: the story so far. Nat Rev Neurol.

[69] Farris W, Mansourian S, Chang Y, Lindsley L, Eckman EA, Frosch MP, Eckman CB, Tanzi RE, Selkoe DJ, Guenette S (2003). Insulin-degrading enzyme regulates the levels of insulin, amyloid beta-protein, and the beta-amyloid precursor protein intracellular domain in vivo. Proc Natl Acad Sci U S A.

[70] Donahue JE, Flaherty SL, Johanson CE, Duncan JA, Silverberg GD, Miller MC (2006). RAGE, LRP-1, and amyloid-beta protein in Alzheimer’s disease. Acta Neuropathol.

[71] Arendt T (2009). Synaptic degeneration in Alzheimer’s disease. Acta Neuropathol.

[72] Calon F, Lim GP, Yang F, Morihara T, Teter B, Ubeda O, Rostaing P, Triller A, Salem N, Ashe KH, Frautschy SA, Cole GM (2004). Docosahexaenoic acid protects from dendritic pathology in an Alzheimer’s disease mouse model. Neuron..

[73] Julien C, Tremblay C, Bendjelloul F, Phivilay A, Coulombe M-A, Emond V (2008). Decreased drebrin mRNA expression in Alzheimer disease: correlation with tau pathology. J Neurosci Res.

[74] Kalaria RN, Harik SI (1989). Reduced glucose transporter at the blood-brain barrier and in cerebral cortex in Alzheimer disease. J Neurochem.

[75] Simpson IA, Chundu KR, Davies-Hill T, Honer WG, Davies P (1994). Decreased concentrations of GLUT1 and GLUT3 glucose transporters in the brains of patients with Alzheimer’s disease. Ann Neurol.

[76] Liu Y, Liu F, Iqbal K, Grundke-Iqbal I, Gong C-X (2008). Decreased glucose transporters correlate to abnormal hyperphosphorylation of tau in Alzheimer disease. FEBS Lett.

[77] Nicholson RM, Kusne Y, Nowak LA, LaFerla FM, Reiman EM, Valla J (2010). Regional cerebral glucose uptake in the 3xTG model of Alzheimer’ disease highlights common regional vulnerability across AD mouse models. Brain Res.

[78] Do TM, Alata W, Dodacki A, Traversy M-T, Chacun H, Pradier L, Scherrmann JM, Farinotti R, Calon F, Bourasset F (2014). Altered cerebral vascular volumes and solute transport at the blood-brain barriers of two transgenic mouse models of Alzheimer’ disease. Neuropharmacology..

[79] Do TM, Dodacki A, Alata W, Calon F, Nicolic S, Scherrmann J-M (2016). Age-dependent regulation of the blood-brain barrier influx/efflux equilibrium of amyloid-β peptide in a mouse model of Alzheimer’s disease (3xTg-AD). J Alzheimers Dis.

[80] Bourasset F, Ouellet M, Tremblay C, Julien C, Do TM, Oddo S, LaFerla F, Calon F (2009). Reduction of the cerebrovascular volume in a transgenic mouse model of Alzheimer’s disease. Neuropharmacology..

[81] Loh RKC, Formosa MF, La Gerche A, Reutens AT, Kingwell BA, Carey AL. Acute metabolic and cardiovascular effects of mirabegron in healthy individuals. Diabetes Obes Metab. 2019;21(2):276–84. 10.1111/dom.13516. 10.1111/dom.13516 30203462

[82] Nedergaard J, Cannon B (2013). UCP1 mRNA does not produce heat. Biochim Biophys Acta.

[83] Harms M, Seale P (2013). Brown and beige fat: development, function and therapeutic potential. Nat Med.

[84] Adebakin A, Bradley J, Gümüsgöz S, Waters EJ, Lawrence CB (2012). Impaired satiation and increased feeding behaviour in the triple-transgenic Alzheimer’s disease mouse model. PLoS One.

[85] Do K, Laing BT, Landry T, Bunner W, Mersaud N, Matsubara T (2018). The effects of exercise on hypothalamic neurodegeneration of Alzheimer’s disease mouse model. PLoS One.

[86] Gavrilova O, Marcus-Samuels B, Reitman ML (2000). Lack of responses to a beta3-adrenergic agonist in lipoatrophic A-ZIP/F-1 mice. Diabetes..

[87] Szentirmai É, Kapás L (2017). The role of the brown adipose tissue in β3-adrenergic receptor activation-induced sleep, metabolic and feeding responses. Sci Rep.

[88] Griebling TL, Campbell NL, Mangel J, Staskin D, Herschorn S, Elsouda D, Schermer CR (2020). Effect of mirabegron on cognitive function in elderly patients with overactive bladder: MoCA results from a phase 4 randomized, placebo-controlled study (PILLAR). BMC Geriatr.

[89] Gibbs ME, Maksel D, Gibbs Z, Hou X, Summers RJ, Small DH (2010). Memory loss caused by beta-amyloid protein is rescued by a beta(3)-adrenoceptor agonist. Neurobiol Aging.

[90] Gunstad J, Lhotsky A, Wendell CR, Ferrucci L, Zonderman AB (2010). Longitudinal examination of obesity and cognitive function: results from the Baltimore longitudinal study of aging. Neuroepidemiology.

[91] Takeda S, Sato N, Uchio-Yamada K, Sawada K, Kunieda T, Takeuchi D, Kurinami H, Shinohara M, Rakugi H, Morishita R (2010). Diabetes-accelerated memory dysfunction via cerebrovascular inflammation and Abeta deposition in an Alzheimer mouse model with diabetes. Proc Natl Acad Sci U S A.

[92] Abbondante S, Baglietto-Vargas D, Rodriguez-Ortiz CJ, Estrada-Hernandez T, Medeiros R, LaFerla FM (2014). Genetic ablation of tau mitigates cognitive impairment induced by type 1 diabetes. Am J Pathol.

[93] Rajasekar N, Nath C, Hanif K, Shukla R (2017). Intranasal insulin improves cerebral blood flow, Nrf-2 expression and BDNF in STZ (ICV)-induced memory impaired rats. Life Sci.

[94] Tong X-K, Trigiani LJ, Hamel E (2019). High cholesterol triggers white matter alterations and cognitive deficits in a mouse model of cerebrovascular disease: benefits of simvastatin. Cell Death Dis.

[95] Evans BA, Papaioannou M, Bonazzi VR, Summers RJ (1996). Expression of beta 3-adrenoceptor mRNA in rat tissues. Br J Pharmacol.

[96] Summers RJ, Papaioannou M, Harris S, Evans BA (1995). Expression of beta 3-adrenoceptor mRNA in rat brain. Br J Pharmacol.

[97] Stemmelin J, Cohen C, Terranova J-P, Lopez-Grancha M, Pichat P, Bergis O, Decobert M, Santucci V, Françon D, Alonso R, Stahl SM, Keane P, Avenet P, Scatton B, le Fur G, Griebel G (2008). Stimulation of the beta3-Adrenoceptor as a novel treatment strategy for anxiety and depressive disorders. Neuropsychopharmacology.

[98] Consoli D, Leggio GM, Mazzola C, Micale V, Drago F (2007). Behavioral effects of the beta3 adrenoceptor agonist SR58611A: is it the putative prototype of a new class of antidepressant/anxiolytic drugs?. Eur J Pharmacol.

[99] Tamburella A, Micale V, Leggio GM, Drago F (2010). The beta3 adrenoceptor agonist, amibegron (SR58611A) counteracts stress-induced behavioral and neurochemical changes. Eur Neuropsychopharmacol.

[100] Schneider LS, Sano M (2009). Current Alzheimer’ disease clinical trials: methods and placebo outcomes. Alzheimers Dement.

[101] O'Connor A, Pannee J, Poole T, Arber C, Portelius E, Swift IJ, et al. Plasma amyloid-β ratios in autosomal dominant Alzheimer’ disease: the influence of genotype. Brain. 2021. 10.1093/brain/awab166. 10.1093/brain/awab166 PMC8634092 33892504

[102] Sproul AA, Jacob S, Pre D, Kim SH, Nestor MW, Navarro-Sobrino M, Santa-Maria I, Zimmer M, Aubry S, Steele JW, Kahler DJ, Dranovsky A, Arancio O, Crary JF, Gandy S, Noggle SA (2014). Characterization and molecular profiling of PSEN1 familial Alzheimer’ disease iPSC-derived neural progenitors. PLoS One.

[103] Kumar-Singh S, Theuns J, Van Broeck B, Pirici D, Vennekens K, Corsmit E (2006). Mean age-of-onset of familial Alzheimer disease caused by presenilin mutations correlates with both increased Abeta42 and decreased Abeta40. Hum Mutat.

[104] Tanzi RE (2012). The genetics of Alzheimer disease. Cold Spring Harb Perspect Med.

[105] Jacobsen JS, Wu C-C, Redwine JM, Comery TA, Arias R, Bowlby M, Martone R, Morrison JH, Pangalos MN, Reinhart PH, Bloom FE (2006). Early-onset behavioral and synaptic deficits in a mouse model of Alzheimer’s disease. Proc Natl Acad Sci U S A.

[106] Arber C, Toombs J, Lovejoy C, Ryan NS, Paterson RW, Willumsen N (2019). Familial Alzheimer’ disease patient-derived neurons reveal distinct mutation-specific effects on amyloid beta. Mol Psychiatry.

[107] Toombs J, Foiani MS, Wellington H, Paterson RW, Arber C, Heslegrave A (2018). Amyloid β peptides are differentially vulnerable to preanalytical surface exposure, an effect incompletely mitigated by the use of ratios. Alzheimers Dement (Amst).

[108] Oblak AL, Forner S, Territo PR, Sasner M, Carter GW, Howell GR (2020). Model organism development and evaluation for late-onset Alzheimer’s disease: MODEL-AD. Alzheimers Dement Transl Res Clin Interv.

[109] Finlin BS, Memetimin H, Zhu B, Confides AL, Vekaria HJ, el Khouli RH, Johnson ZR, Westgate PM, Chen J, Morris AJ, Sullivan PG, Dupont-Versteegden EE, Kern PA (2020). The β3-adrenergic receptor agonist mirabegron improves glucose homeostasis in obese humans. J Clin Invest.

[110] Hainer V (2016). Beta3-adrenoreceptor agonist mirabegron – a potential antiobesity drug?. Expert Opin Pharmacother.

[111] Weyer C, Tataranni PA, Snitker S, Danforth E, Ravussin E (1998). Increase in insulin action and fat oxidation after treatment with CL 316,243, a highly selective beta3-adrenoceptor agonist in humans. Diabetes..

[112] Chapple CR, Kaplan SA, Mitcheson D, Blauwet MB, Huang M, Siddiqui E (2014). Mirabegron 50 mg once-daily for the treatment of symptoms of overactive bladder: an overview of efficacy and tolerability over 12 weeks and 1 year. Int J Urol.

[113] Chapple CR, Siddiqui E (2017). Mirabegron for the treatment of overactive bladder: a review of efficacy, safety and tolerability with a focus on male, elderly and antimuscarinic poor-responder populations, and patients with OAB in Asia. Expert Rev Clin Pharmacol.

[114] Herschorn S, Staskin D, Schermer CR, Kristy RM, Wagg A (2020). Safety and tolerability results from the PILLAR study: a phase iv, double-blind, randomized, placebo-controlled study of Mirabegron in patients ≥ 65 years with overactive bladder-wet. Drugs Aging.

