# Supplementary figures and images for: Repurposing beta-3 adrenergic receptor agonists for Alzheimer’s disease: beneficial effects in a mouse model
Source: Alzheimers Res Ther. 2021 May 21;13:103. doi: 10.1186/s13195-021-00842-3 (PMC8140479; doi:10.1186/s13195-021-00842-3)

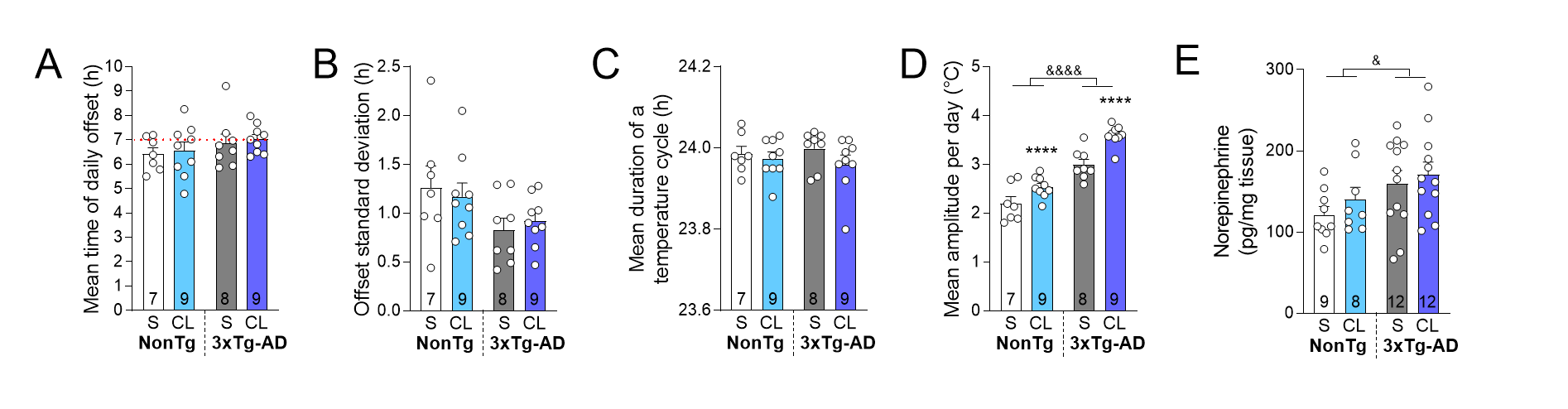

Supplement: Supplementary file 1 — Additional file 1. CL-316,243 administration does not affect circadian rhythm parameters nor BAT norepinephrine content. A: individual mean daily offsets (determined as the time of the first six successive bins when temperature was lower than the mean diurnal temperature, thus corresponding to morning temperature drop). B: Offset standard deviation. C: Mean duration of a total temperature cycle. D: Mean amplitude of body temperature during one day (24-h, from 7 a.m. to 7 p.m.). E: Norepinephrine concentrations measured by HPLC in BAT, normalized to tissue weights. Data are represented as mean ± SEM (n/group indicated in bars). Statistics: Two-way ANOVA, effect of CL-316,243 treatment: ****p < 0.0001, effect of genotype: &p < 0.05 &&&&p < 0.0001 (A-E). Abbreviations: 3xTg-AD: triple transgenic mice; CL: CL-316,243-injected group; NonTg: non-transgenic mice; S: saline-injected group [file 13195_2021_842_MOESM1_ESM.tif]
